# Left Ventricular Assist Device Multialarm Emergency: A High-Fidelity Simulation Case for Emergency Medicine Residents

**DOI:** 10.15766/mep_2374-8265.11156

**Published:** 2021-05-05

**Authors:** Ryan Barnicle, Sean Boaglio, Jillian Fitzgerald, Karalynn Otterness, Scott Johnson, Christine Ahn

**Affiliations:** 1 Clinical Instructor, Department of Emergency Medicine, Stony Brook University Hospital; 2 Clinical Instructor, Department of Emergency Medicine, Vanderbilt University Medical Center; 3 LVAD Coordinator, Department of Cardiothoracic Surgery, Stony Brook University Hospital; 4 Assistant Residency Program Director, Department of Emergency Medicine, Stony Brook University Hospital; 5 Residency Program Director, Department of Emergency Medicine, Stony Brook University Hospital

**Keywords:** Emergency Medicine, Left Ventricular Assist Device, LVAD, Simulation, Sepsis, Shock, Cardiac Arrest

## Abstract

**Introduction:**

As left ventricular assist devices (LVADs) become more prevalent in the treatment of patients with end-stage heart failure, emergency physicians must become experts in the management and resuscitation of patients with LVADs. As with other high-acuity, low-occurrence scenarios, managing the unstable LVAD patient makes for an ideal topic for simulation-based resident education.

**Methods:**

By incorporating a high-fidelity HeartMate 3 LVAD task trainer, our program developed and executed a novel LVAD simulation activity for our emergency medicine resident physicians. In the scenario, a 65-year-old male with recent LVAD placement arrived at a community hospital with undifferentiated hypotension. Various device alarms activated during the scenario and required intervention. Ultimately, the patient was found to be in septic/hypovolemic shock and only survived with appropriate resuscitation. We implemented a postscenario survey to assess the effectiveness of the simulation activity and administered it to 27 residents.

**Results:**

Content and delivery of our simulation were found to be effective; all survey questions regarding content and delivery obtained a mean score of 4.5 or greater on a 5-point Likert scale. Residents reported an overall high level of confidence in achieving most of the skill-based learning objectives (most scores > 4.1). The two objectives with the lowest confidence ratings were troubleshooting an LVAD and its various alarms (3.8) and demonstrating the ability to assess an LVAD patient (3.9).

**Discussion:**

Our LVAD simulation activity was successful and also revealed several potential areas for future research and simulation improvement.

## Educational Objectives

By the end of the simulation exercise, all learners will be able to:

1. Demonstrate a systematic approach to evaluating a patient with a left ventricular assist device (LVAD).
2. Attain an accurate blood pressure in a patient with an LVAD.
3. Differentiate and rectify various LVAD alarms, including impending power failure.
4. Formulate a differential diagnosis for LVAD low-flow alarms.
5. Diagnose and manage septic/hypovolemic shock in a patient with an LVAD.
6. Summarize a concise and relevant report to the LVAD consultant team.
7. Appropriately employ advanced cardiovascular life support for a patient with an LVAD in cardiac arrest.

## Introduction

Heart failure is very common in the United States and is associated with a significant number of emergency department (ED) presentations and subsequent inpatient admissions each year. Patients with end-stage disease are increasingly qualifying for a left ventricular assist device (LVAD) once they meet certain diagnostic or therapeutic criteria^1^ in order to facilitate a bridge to transplant or a bridge to recovery, or as destination therapy. As expected, the number of LVAD patients presenting to the ED with LVAD-related, LVAD-associated, and general medical issues is increasing. Although the overall current number of US presentations is not known, one major medical center with an affiliated LVAD program performed a retrospective chart review of all patients who had received an LVAD implantation from 2011 to 2015 and found a rate of 3.49 visits per patient during the study period.^2^ According to the Centers for Medicare & Medicaid Services, there are 183 facilities offering VAD destination therapy, many of which have associated emergency medicine (EM) residencies.^3^

Emergency physicians are now expected to know how to provide initial assessments and interventions for these patients, and it is imperative that they coordinate consultation with the designated ventricular assist device (VAD) management team for both routine and critical presentations. In fact, the management of LVADs was added to the 2016 Model of the Clinical Practice of Emergency Medicine and remains in the newly released 2019 version.^4^ Specific mean arterial pressure (MAP) goals make hemodynamic management challenging, and the devices will alarm with excessive deviations in preload or afterload. Gastrointestinal bleeding is a very common problem for these patients due to the development of arteriovenous malformations and is further complicated by their requisite anticoagulation with warfarin. Strokes, dysrhythmias, and right ventricular failure are also frequent presentations. Device-specific suction events, thrombosis, and driveline infections must also be considered and managed.

In recent years, several comprehensive review papers have been published that outline systematic approaches to patients presenting to the ED with LVADs and are an excellent resource for addressing the complications above. Trinquero and colleagues provide an overview of the three currently FDA-approved LVADs in use, as well as a detailed ED algorithm for managing patients with LVADs, including initial assessment, appropriate diagnostic studies, anticipating complications, and troubleshooting alarms.^5^ Long, Robertson, Koyfman, and Brady offer excellent visual schematics of the HeartMate 3 controller and detailed tables summarizing LVAD complications, alarm notifications, special considerations, and management recommendations.^6^ Their publication serves as a great reference for emergency physicians when treating patients with LVADs and was one of the primary sources used in the design of our simulation case. For those preferring case-based discussion, Robertson, Long, and Koyfman go into detail on managing specific presentations.^7^ Even an unusual case of an accidentally severed driveline emphasizes just how dependent patients are on functioning devices.^8^ These resources universally recommend that chest compressions are indicated for patients in cardiac arrest despite manufacturers’ reservations about causing cannula displacement.

Perhaps the most significant conclusion in the current literature is that “given the evolving technology and increasing prevalence of VADs, the ED community would benefit from… VAD-specific training programs in residency training.”^5^ Since LVAD catastrophes are still high-acuity, low-occurrence (HALO) events even at our institution, our program leaders decided that incorporating one into the curriculum as a simulation activity would be the most effective way to instruct EM residents. There has been extensive research into simulation-based learning in medicine, and some EM residency programs have even trialed converting entire curriculums to a simulation-based format with good reception from residents.^9^ This is not unexpected as a prior study showed that both EM residents and attendings typically shared the accommodating learning style as defined by the Kolb Learning Style Inventory; this style emphasizes active experimentation and concrete experience.^10^ Barlas, Gupta, Lesser, and Tai found EM residents to also prefer experiential learning as defined by Kolb.^11^ This provides theoretical support for using simulation as pedagogy.

Another simulation in *MeEdPORTAL* focuses on an LVAD patient with accidental multitrauma,^12^ while our case is a medical scenario that uses a high-fidelity task trainer nearly identical to our institution's first-line HeartMate 3. A more recently published simulation scenario emphasizes an impressively moulaged SimMan 3G, but the added components are meant only to imitate an LVAD, not replicate one.^13^ To our knowledge, ours is the first simulation case for EM residents to utilize a fully functioning LVAD HeartMate 3 task trainer.

## Methods

### Development

Our EM residents received an overview from our institution's LVAD coordinators regarding the preferred institution-specific ED workup and management of patients presenting with LVADs. (A version of that presentation is included here for reference only as Appendix A.) Given the increasing frequency of these presentations and the new inclusion in the national curriculum, it was decided that incorporating a patient with an alarming LVAD into the simulation curriculum was imperative. In order to maximize authenticity, we utilized a high-fidelity, fully functional HeartMate 3 task trainer (Abbott; see the first figure in Appendix B) borrowed from the Cardiothoracic Intensive Care Unit (CTICU) and connected to our SimMan 3G computerized manikin (Laerdal; see the second and third figures in Appendix B).

This simulation case was developed for EM residents of all levels of training. While prerequisite knowledge was not required to participate, most of our residents had received an overview from the LVAD coordinator at our hospital. This overview included an explanation of how heart failure patients qualify for LVAD implantation, essential labs to order in the ED, how to accurately assess blood pressure, interpretation of the speed/power/flow controller displays, hazard and advisory alarm management, driveline infection assessment, LVAD anticoagulation guidelines, common LVAD complications, and cardiac arrest management. There was no assigned prereading. We did a literature review before designing the case and found that our institution-specific recommendations were largely consistent with the broader literature. One exception was that our LVAD coordinators asked that driveline sites be left sterile and that EM providers defer inspection to them since there was always one available on call. This practice was in contrast to the literature, which noted it as a significant source of sepsis, and thus, we incorporated driveline site inspection into our scenario.

### Equipment/Environment

The simulation scenario (Appendix C) was conducted in our hospital's simulation center, which had two rooms resembling our ED resuscitation bays. All standard monitoring equipment was available and was connected to our SimMan 3G. A fully functional HeartMate 3 LVAD controller with a task trainer that pumped water (simulating blood flow) through a closed tubing circuit was inserted beneath the torso skin and the chest plate of the manikin to simulate authentic implantation complete with the palpable hum of a functioning LVAD (see the third figure in Appendix B). Also, the functioning controller capable of alarming and interrogation was attached via an embedded abdominal driveline (see the second figure in Appendix B). Additional equipment (outlined in the simulation case template in Appendix C) included a monitor capable of measuring continuous ECG, noninvasive blood pressure, pulse oximetry, temperature, arterial blood pressure, and end-tidal carbon dioxide. Defibrillator pads and a vascular doppler probe were at the bedside. Gloves, masks, gowns, nasal cannula, IV lines, a non-rebreather mask, an arterial line kit, IV meds/code cart, standard airway equipment, a bag-valve mask, sterile dressing kit, and phone were also made available. Case stimuli included a chest X-ray, ECG, point-of-care ultrasound clips, and lab values as listed in Appendix D.

### Personnel

One of our academic chief residents was available for each run-through to play the role of the paramedic giving report, the wife giving background information, and the prompting nurse if needed. The chief resident remained present to subtly manipulate the LVAD loop to induce low-flow alarms by applying pressure to the closed loop, which triggered the low-flow alarm by temporarily inhibiting forward flow of water. An EM attending was in the control room and played the role of the LVAD consult service. Several of our simulation center staff members were integral as always in setting up the resuscitation rooms.

### Implementation

Twenty-seven EM residents participated in this simulation exercise, divided into six groups. Four subintern medical students also observed the cases. The exercise occurred at one of our monthly simulation sessions during protected resident education time. A senior resident was designated team leader; the team leader then assigned other residents the roles for various anticipated tasks (airway management, vascular access, vital signs and physical examination, point-of-care ultrasound, ECG acquisition, and medication administration). The case involved a 62-year-old man who recently had an LVAD implantation presenting to the ED via emergency medical services with nausea, vomiting, diarrhea, and lethargy. Each resuscitation scenario ran for about 20 minutes, with roughly 10 minutes of debriefing. At the conclusion of the case, the critical actions (Appendix E) were reviewed with the participants. The EM attending then completed a debriefing (Appendix F). Following the simulation, a survey (Appendix G) was sent to residents to determine the effectiveness of the educational activities.

### Debriefing

The case was debriefed by the EM attending running the simulation using a three-phase debriefing technique described by Rudolph, Simon, Raemer, and Eppich.^14^ The first phase—reaction—allowed trainees to express immediate initial reactions, both negative and positive, to the simulation scenario. This was followed by an analysis phase where instructors could help learners identify performance gaps as compared to predetermined critical actions and objectives. The attending had a list of sample questions to facilitate a discussion format over pure didactic lecture during the debriefing. Finally, a summary phase occurred where the attending was able to deliver the most salient takeaways that we deemed important for learners to internalize.

All of the participants had the opportunity to inspect and interrogate both the HeartMate 3 pump and the controller. Sample questions used for discussion are included in Appendix F with sample answers. Major takeaways we desired residents to obtain are also explicitly listed in Appendix F. Algorithms modified from Trinquero and colleagues^5^ regarding the general ED approach to the VAD patient and the specific approach to the low-flow alarm were available to the EM attendings conducting the debriefing. An overview table regarding LVAD conditions and complications from Long, Robertson, Koyfman, and Brady^6^ was also used. Both were made available to the residents and are also included here in Appendix F for reference.

### Assessment

During the simulation, the following critical actions (Appendix E) were monitored by the supervising EM attending:

• Confirming the LVAD was functioning by assessing for a thrum under the chest wall;
• Obtaining a reliable blood pressure with either doppler or arterial line;
• Identifying a fever via core temperature;
• Ensuring a reliable source of power when the battery alarm started going off;
• Treating septic shock and hypovolemia empirically, once they had been identified as the likely source of the low-flow alarm, with antibiotics and fluid resuscitation and vasopressors;
• Inspecting the driveline insertion site in sterile fashion;
• Giving appropriate chest compressions during cardiac arrest; and
• Performing intubation in a hemodynamically neutral fashion.

These actions were LVAD specific in addition to general resuscitation care.

A simulation evaluation tool (Appendix G) using 5-point Likert scales was distributed to all participants to determine the effectiveness of the simulation exercise in meeting its educational objectives. All 27 residents completed the simulation activity and then provided feedback by filling out an anonymous survey during the following week's conference. Participants were asked to rate their answers using a 5-point Likert scale (1 = *Strongly Disagree,* 5 = *Strongly Agree*) in order to evaluate the case content and its effectiveness. Participants also responded to questions assessing their confidence (1 = *Very Unconfident,* 5 = *Very Confident*) with skill-based learning objectives. Participant surveys included a component for subjective feedback regarding the simulation experience, educational content, and suggestions for improvement.

## Results

The data show that the participating residents regarded the content and the delivery of the simulation positively. All of the survey statements for content and delivery ranged over 4.5 out of 5, correlating with Strongly Agree for each, as outlined in Table 1. Standard deviations were 0.8 for all statements (see Table 1). It should be noted that there was a single respondent who selected 1 for all responses. Given that this was a significant deviation from all other respondents, we have high suspicion it was a reverse-interpretation of the rating scale, especially because that particular respondent scored the statements in the second half of the survey positively. Regardless, this participant was not removed from the calculations. Overall, the responses show that the residents considered the scenario relevant, realistic, and effective. The responses also show that the debrief was deemed reflective, helpful, and done in a safe environment.

**Table 1.**
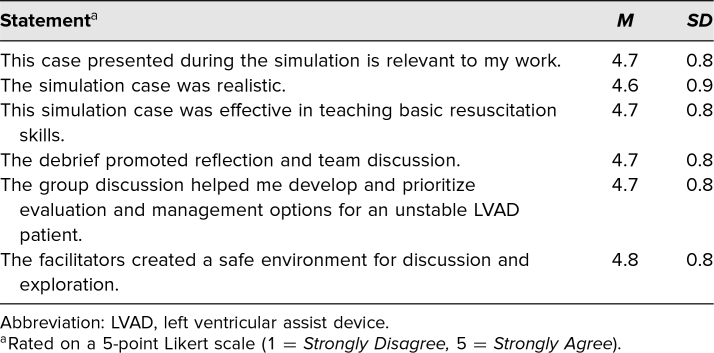
Scores for Evaluation of Content and Delivery

Table 2 highlights that the learning objectives were successfully achieved. The statements that received the lowest two average scores were “Demonstrate the ability to assess an LVAD patient” and “Troubleshoot an LVAD and its various alarms,” scoring 3.9 and 3.8, respectively, which correlate with Neutral comfort levels. All other statements correlate with Confident comfort levels.

**Table 2.**
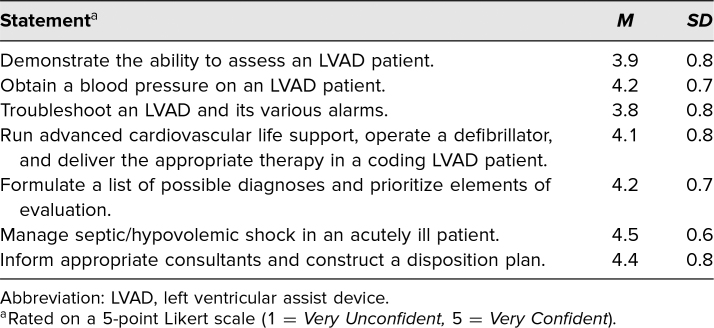
Scores for Confidence With Learning Objectives After Participating in the LVAD Simulation Case

## Discussion

Traditionally, LVAD complications have been considered HALO events. With a rise in facilities offering LVADs as definitive therapy for advanced heart failure, patients with implanted LVADs are becoming commonplace in EDs across the country. Since patients visit their defined LVAD centers and community EDs alike, all emergency physicians must be knowledgeable in caring for patients with LVADs. Through our described simulation, we have shown that high-fidelity LVAD patient simulation is both feasible and efficient in its delivery of educational content as well as being an effective method for providers to gain confidence managing this unique patient population.

The construction of LVAD simulation models has previously been described, utilizing materials to mimic a functioning LVAD device in conjunction with a simulation manikin.^13^ Our presented model utilizing a HeartMate 3 task trainer embedded under the chest plate of a SimMan 3G manikin demonstrates a higher-fidelity version allowing for authentic palpation of the thrum of a functioning LVAD, realistic interaction with the LVAD power supply, and visualization of alarms and settings on the device controller identical to those most frequently encountered at our tertiary medical center. We theorize that most LVAD centers likely have task trainers for nursing and patient education, often in their CTICUs, that are potentially available for interdisciplinary use.

As shown in Table 1, all aspects pertaining to satisfaction with educational content and its delivery produced scores averaging greater than 4.5. This indicates the simulation model was regarded by trainees as a relevant, realistic, and effective teaching modality that allowed for meaningful group debriefing to solidify learned concepts. As seen in Table 2, the breakdown of participants’ confidence in specific learning objectives indicates the importance of continued utilization of this LVAD training exercise in the simulation curriculum. Survey elements of “Demonstrate the ability to assess an LVAD patient” and “Troubleshoot an LVAD and its various alarms” averaged the lowest scores, at 3.9 and 3.8, respectively. The fact that these elements scored lower than others indicates future potential for modifying the simulation activity to include multiple case variations, perhaps performed in rapid succession, each variation emphasizing different LVAD alarms and associated complications. Repeated exposure to LVAD simulation activities could address participants’ lack of confidence with LVAD functionality and troubleshooting the multiple alarms. A high level of confidence was achieved when it came to hemodynamic assessment, performance of cardiopulmonary resuscitation, formulation of differential diagnosis, initiation of appropriate consultation, and construction of a disposition plan for the LVAD patient, as indicated in Table 2 by Likert-scale scores all averaging greater than 4.1 for the other key learning objectives. These results highlight the simulation's ability to teach key learning objectives of managing LVAD patients in the ED and are an important first step toward skill mastery in this otherwise HALO patient population. Further consideration of implementing LVAD simulation activities in the residency program curriculum on a recurring basis is warranted.

In their comments, residents stated they felt the simulation was useful because it allowed them to become more familiar with the LVAD equipment and troubleshoot common LVAD alarms. They learned how to perform basic maneuvers such as hooking up the LVAD to an external power supply and swapping batteries, as well as the importance of using arterial lines and doppler MAPs to evaluate blood pressure for sick LVAD patients. Residents felt the simulation could be improved by having fewer participants per group to facilitate closed-loop communications and possibly by pairing the simulation with a lecture and handout. Multiple residents asked for the simulation to be given more frequently to help them become more comfortable with such a unique population of patients.

Our investigation has some limitations that warrant further discussion. We included only 27 residents from our own training program, and therefore, our results may not be broadly applicable across different populations and may require external validation. Future research could yield more powerful and generalizable results by including additional participants. Participating resident physicians completed a postsimulation survey to evaluate the educational content and its delivery, as well as their confidence with the learning objectives. Educational impact of this simulation could be more accurately assessed if both pre- and postcompletion surveys were utilized. Additionally, as is the case with many survey studies, our results may have been affected by recall bias since the survey was completed a week after the simulation session. These limitations indicate the need for ongoing evaluation of the impact of high-fidelity LVAD-associated simulation activities.

### Conclusion

Overall, results from the survey suggest that the content and delivery of our LVAD simulation activity were successful, as evidenced by high scores on the 5-point Likert scales. Additionally, after participating in the simulation activity, our residents rated overall high levels of confidence in most of the skill-based learning objectives. The areas scoring the lowest (ability to assess an LVAD patient and troubleshoot the LVAD and its various alarms) provide valuable information by highlighting topics that will require additional emphasis in our curriculum in the future, which is more than reasonable given the complexity of these patients. Our scenario covers only a narrow aspect of the many LVAD complications that can occur, and multiple cases can be devised that incorporate this high-fidelity setup to expose residents to these complex patients.

## Appendices

Institutional LVAD Coordinator Educational Presentation.pptxHeartMate 3 Task Trainer Setup.docxSimulation Case.docxSimulation Images.docxCritical Actions.docxDebriefing Materials.docxSurvey.docx
All appendices are peer reviewed as integral parts of the Original Publication.
