# Supplementary material for: Left Ventricular Assist Device Multialarm Emergency: A High-Fidelity Simulation Case for Emergency Medicine Residents
Source: MedEdPORTAL. 2021 May 5;17:11156. doi: 10.15766/mep_2374-8265.11156 (PMC8096883; doi:10.15766/mep_2374-8265.11156)
Supplement: Supplementary file 1 — Institutional LVAD Coordinator Educational Presentation.pptxHeartMate 3 Task Trainer Setup.docxSimulation Case.docxSimulation Images.docxCritical Actions.docxDebriefing Materials.docxSurvey.docx [file mep_2374-8265.11156-s001.zip › D. Simulation Images.docx]

Appendix D. Simulation Images

ECG


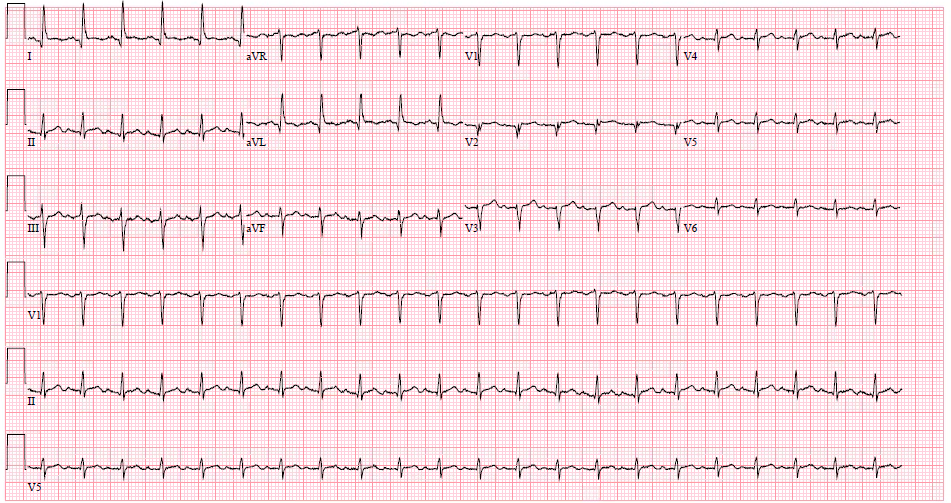


Citation: Author Owned

Chest X-ray


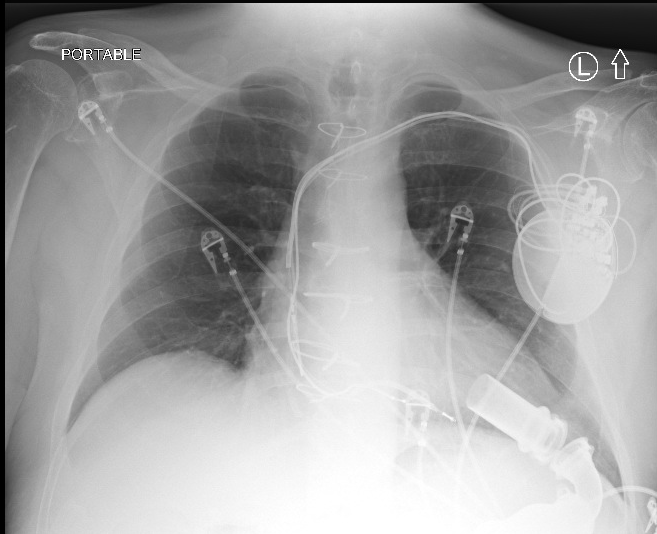


Citation: Author Owned

IVC


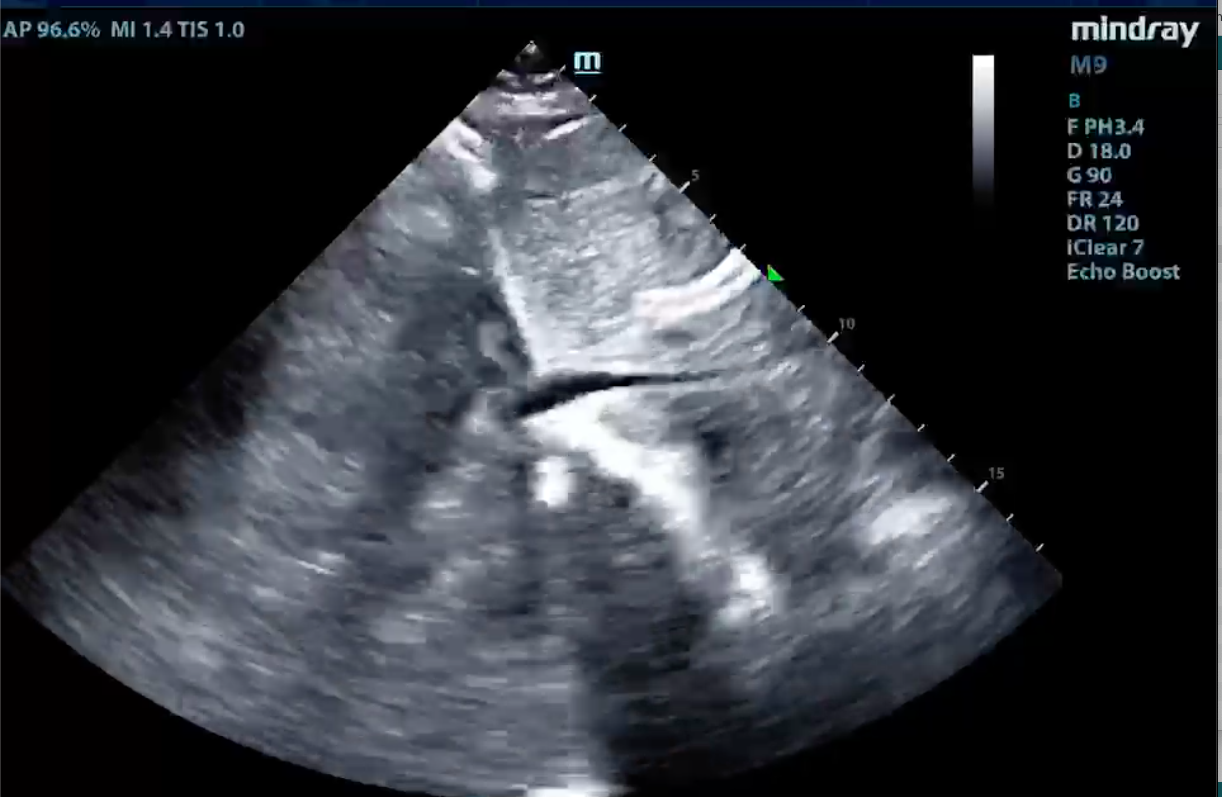


Citation: Author Owned

Apical 4 Chamber POCUS View


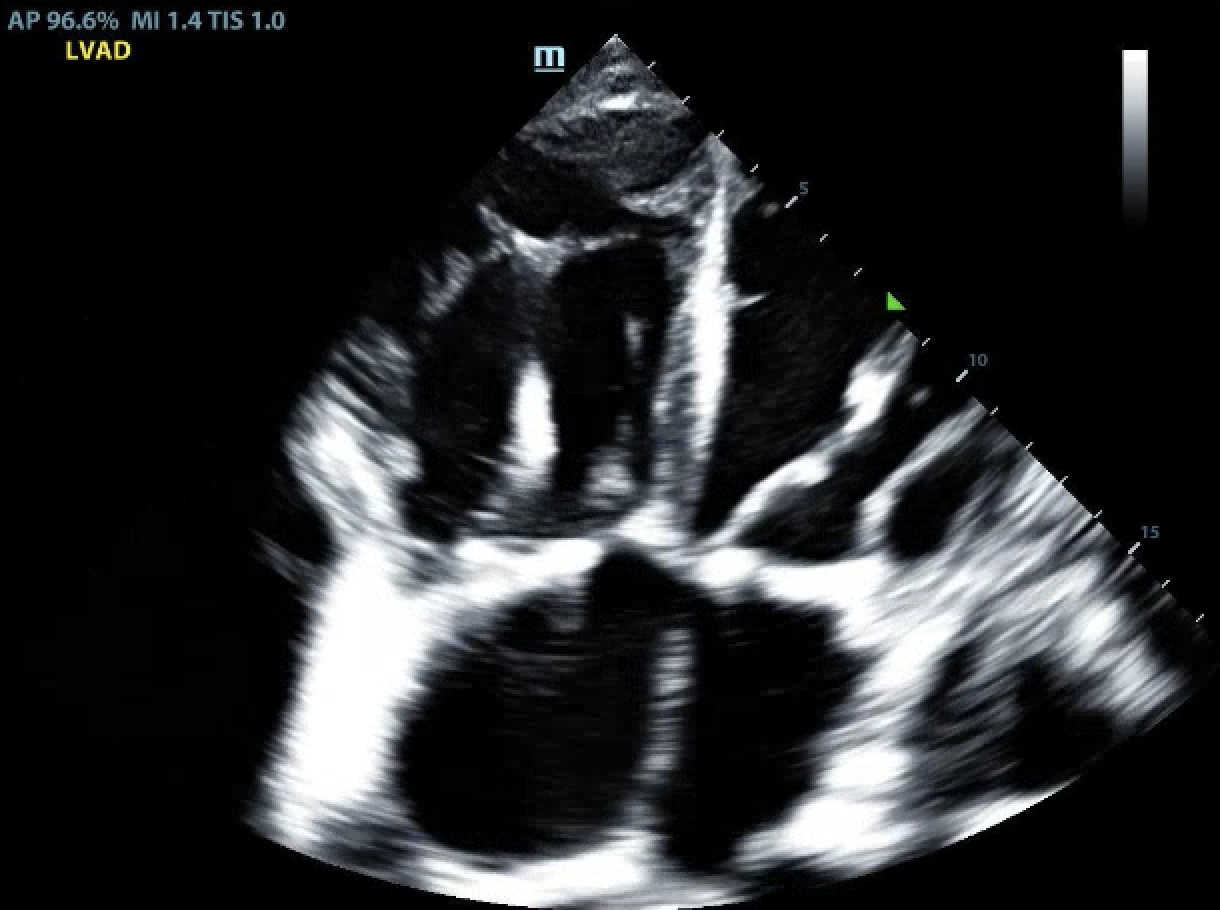


Citation: Author Owned

Laboratory Values

| TEST | RESULT | REFERENCE RANGE |
| --- | --- | --- |
| COMPLETE BLOOD COUNT |  |  |
| WBC | 16.2 K/uL | (4.8-10.8 K/uL) |
| Hemoglobin | 10.5 g/dL | (14.0-18.0 g/dL) |
| Hematocrit | 38% | (42-52%) |
| Platelets | 345 K/uL | (150-350 K/uL) |
| BASIC METABOLIC PROFILE |  |  |
| Sodium | 134 mmol/L | (135-146 mmol/L) |
| Chloride | 108 mmol/L | (96-107 mmol/L) |
| Potassium | 5.6 mmol/L | (3.5-5.1 mmol/L) |
| Bicarbonate | 10 mmol/L | (21-31 mmol/L) |
| BUN | 40 mg/dL | (5-20 mg/dL) |
| Creatinine | 3.02 mg/dL | (0.50-1.20 mg/dL) |
| Glucose | 165 mg/dL | (70-99 mg/dL) |
| Calcium | 8.2 mg/dL | (8.6-10.2 mg/dL) |
| TROPONIN | 0.17 ng/mL | (< 0.01 ng/mL) |
| BRAIN NATRIURETIC PEPTIDE (BNP) | 4020 pg/mL | (0-450 pg/mL) |
| LACTATE | 5.2 mmol/L | (0.5-2.0 mmol/L) |
| LACTATE DEHYDROGENASE (LDH) | 220 IU/L | (94-250 IU/L) |
| INTERNATIONAL NORMALIZED RATIO (INR) | 2.6 | (0.8-1.2) |
