# Supplementary material for: Left Ventricular Assist Device Multialarm Emergency: A High-Fidelity Simulation Case for Emergency Medicine Residents
Source: MedEdPORTAL. 2021 May 5;17:11156. doi: 10.15766/mep_2374-8265.11156 (PMC8096883; doi:10.15766/mep_2374-8265.11156)
Supplement: Supplementary file 1 — Institutional LVAD Coordinator Educational Presentation.pptxHeartMate 3 Task Trainer Setup.docxSimulation Case.docxSimulation Images.docxCritical Actions.docxDebriefing Materials.docxSurvey.docx [file mep_2374-8265.11156-s001.zip › E. Critical Actions.docx]

Appendix E. Critical Actions

Date:

Examiner:

Examinee:

Scoring in accordance with the Standardized Direct Observational Tool (SDOT)

The learner(s) should be scored (based on the level of training) for each item below with one of the following:

NI = Needs improvement

ME = Meets Expectations

AE = Above Expectations

NA = Not Assessed

| Critical Action | NI | ME | AE | NA | Category |
| --- | --- | --- | --- | --- | --- |
| Confirm LVAD is functioning by assessing for “thrum” under chest wall |  |  |  |  | PC, MK |
| Obtain a reliable blood pressure with either doppler or arterial line |  |  |  |  | PC, MK |
| Identify a fever via core temperature |  |  |  |  | PC, MK |
| VAD coordinator contact was attempted and reattempted until completed |  |  |  |  | PC, ICS, SBP |
| Ensure a reliable source of power when the battery alarm starts going off |  |  |  |  | PC, MK, PBL |
| Treat septic shock and hypovolemia empirically once identified as the likely source of the low flow alarm with antibiotics and fluid resuscitation and vasopressors |  |  |  |  | PC, MK, PBL |
| Inspect driveline insertion site in sterile fashion |  |  |  |  | PC |
| Give appropriate chest compressions during cardiac arrest |  |  |  |  | PC, MK |
| Perform intubation in a hemodynamically neutral fashion |  |  |  |  | PC, MK, PBL |

Category: One or More of the ACGME Core Competencies as defined in the SDOT

| PC | Patient Care  Compassionate, appropriate, and effective for the treatment of health problems and the promotion of health |
| --- | --- |
| MK | Medical Knowledge  Residents are expected to formulate an appropriate differential diagnosis with special attention to life-threatening conditions, demonstrate the ability to utilize available medical resources effectively, and apply this knowledge to clinical decision making |
| PBL | Practice Based Learning and Improvement  Involves investigation and evaluation of their own patient care, appraisal and assimilation of scientific evidence, and improvements in patient care |
| ICS | Interpersonal Communication Skills  Results in effective information exchange and teaming with patients, their families, and other health professionals |
| P | Professionalism  Manifested through a commitment to carrying out professional responsibilities, adherence to ethical principles, and sensitivity to a diverse patient population |
| SBP | Systems Based Practice  Manifested by actions that demonstrate an awareness of and responsiveness to the larger context and system of health care and the ability to effectively call on system resources to provide care that is of optimal value |
