# Supplementary material for: Left Ventricular Assist Device Multialarm Emergency: A High-Fidelity Simulation Case for Emergency Medicine Residents
Source: MedEdPORTAL. 2021 May 5;17:11156. doi: 10.15766/mep_2374-8265.11156 (PMC8096883; doi:10.15766/mep_2374-8265.11156)
Supplement: Supplementary file 1 — Institutional LVAD Coordinator Educational Presentation.pptxHeartMate 3 Task Trainer Setup.docxSimulation Case.docxSimulation Images.docxCritical Actions.docxDebriefing Materials.docxSurvey.docx [file mep_2374-8265.11156-s001.zip › F. Debriefing Materials.docx]

Appendix F. Debriefing Materials

The case was debriefed by the EM attending running the simulation using a three-phase debriefing technique, specifically utilizing the model of debriefing as described by Rudolph et al.^1^ Rudolf’s three-phrase debriefing technique is comprised of Reaction, Analysis, and Summary.

During the Reaction Phase the faculty facilitator asked the participants “How did you think you performed and how did you feel during the simulation?” This allowed the participants to share their initial reactions and emotions and reflect upon their performance during the simulation in an introspective fashion.

The Analysis Phase of the debriefing session entailed asking specific questions related to the simulation exercise to ascertain any knowledge gaps and prompt discussion between faculty facilitator and participants/learners. Sample questions and pivotal points are included below to assist facilitators. Sample algorithms are also presented for reference in order to assist facilitators regarding clinical information and are modified from published literature. During the Analysis Phase, all of the participants had the opportunity to inspect and interrogate both the HeartMate 3 pump and the controller.

For the Summary Phase, major takeaways are also included. The Summary Phase of the debriefing session drives home the salient points and major takeaways from the simulation session and summarizes what has been learned from the preceding phases.

Sample Questions:

Q: What are reliable ways to obtain a blood pressure in a patient with an LVAD?

A: The Heartmate 3 device is an continuous-flow pump with centrifugal design. This design will create phasic changes in blood flow which create a pulse, although diminished compared with the patient’s native cardiac contraction. Because peripheral and central pulses may be diminished or absent, non-invasive blood pressure measurements may be difficult to obtain. Therefore, the MAP should be obtained by using a BP cuff and doppler ultrasound over the brachial artery. In patients who are critically-ill, blood pressure should be measured with an invasive arterial line. The MAP goal should be 70-80mmHg. Also, don’t forget to use clinical signs of perfusion by assessing the patient’s mental status, skin color and temperature.

Q: What are common LVAD-associated and LVAD-related complications?

A: Arrhythmia, infection (up to 42% become septic within one year-REMATCH study), bleeding (acquired Von Willebrand Disease), PUMP thrombosis, device/pump failure, hypovolemia, RV failure, stroke, GI bleeding

Q: In general, what is the differential diagnosis for both low flow and or high flow alarms?

A: Low flow: pump thrombosis, device failure, acute MI, arrhythmias, hypovolemia, hemorrhage, sepsis, obstructive shock (PE, cardiac tamponade), RV failure. High flow: systemic vasodilation (distributive shock states, medications), liver failure, adrenal failure

Q: Who should be consulted when a patient with an LVAD presents to the emergency department?

A: The hospital’s LVAD coordinator should be contacted immediately. If the hospital does not have an LVAD coordinator, the closest VAD center may be contacted for consultation.

Q: How should cardiac arrest be managed when a patient has an LVAD?

A: Cardiac arrest is managed very similarly to patients without LVADs. Chest compressions should never be withheld out of fear of canulae dislodgement.

Q: What labs should be obtained in a critically ill patient with an LVAD?

A: CBC, PT/PTT/INR, electrolytes, LDH, type and screen. ABGs should be considered to determine hypoxemia as pulse oximeters are usually inaccurate. Troponin levels are not useful in this patient population.

Q: What antibiotics should be used for the empiric treatment of a suspected LVAD-related infection?

A: Choice of antimicrobials for suspected LVAD-related infections should target skin flora and enteric bacteria. In the emergency department, all antimicrobials for these suspected infection should be administered via the intravenous route. Empiric coverage of both *S. aureus* (including methicillin-resistant variants) and *P. aeruginosa* is mandatory. Up to half of these infections will be polymicrobial. One example initial regimen would be a loading dose of 20mg/kg of vancomycin plus 4.5g piperacillin-tazobactam. However, all treating providers should consider local institution guidelines and antimicrobial resistant patterns. For further reading, see Zinoview et al’s review article endorsed by the IDSA.^2^

Pivotal moments in the case:

- Obtaining a reliable blood pressure with BP cuff doppler (MAP) or arterial line
- LVAD coordinator contact was attempted and reattempted if no immediate response
- Ensuring a reliable source of power when the battery alarm started going off.
- Treating septic shock and hypovolemia empirically once identified as the likely source of the low flow alarm- Driveline insertion site was inspected in the sterile fashion

Major takeaways to be emphasized in this case included but were not limited to:

- Patients with LVADs should get immediate consultation with their LVAD coordinators and eventual transfer to tertiary care
- Patients with LVADs are extremely sensitive to both reductions in preload and increases in afterload and both require emergent intervention
- Sepsis is common in LVAD patients. In addition to typical infections, a unique source would be an infected driveline and this should be inspected in sterile fashion, preferably by LVAD team if they are available
- LDH is a lab value that should be ordered for all patients with LVADs
- Cardiac arrest is managed very similarly to patients without LVADs and chest compressions should never be withheld


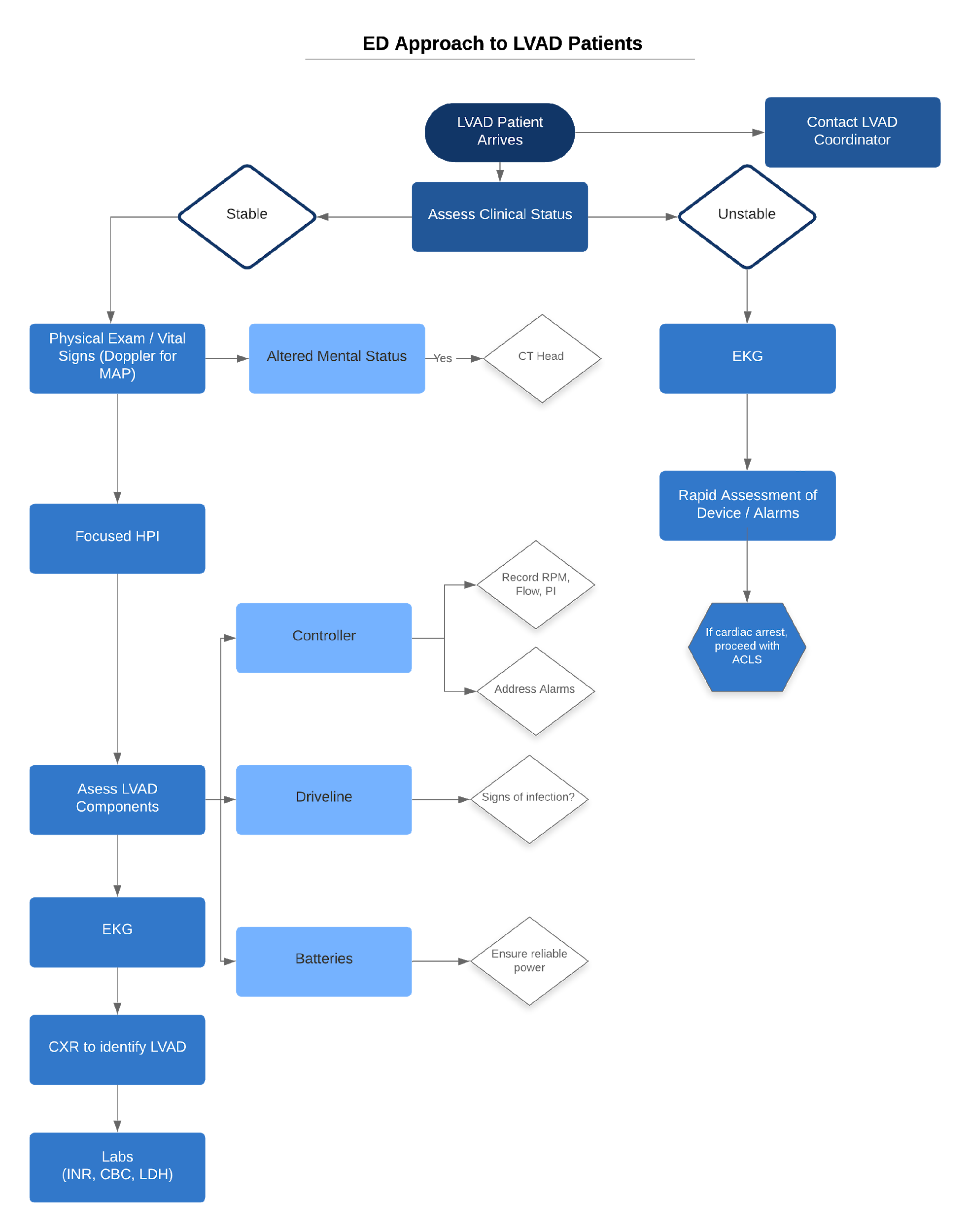


Notes: L*VAD*, left ventricular assist device; *HPI*, history of present illness; *CT*, computed tomography; *EKG*, electrocardiography; *AMS*, altered mental status; *MAP*, mean arterial pressure; *RPM*, revolutions per minute; *PI*, pulsatility index; *PT*, patient; *ACLS*, advanced cardiovascular life support; *CXR*, chest x-ray; *INR,* international normalized ratio; *CBC*, complete blood count; *LDH*, lactic acid dehydrogenase.

Source: Author owned, adapted and modified from [Trinquero P, Pirotte A, Gallagher LP, Iwaki KM, Beach C, Wilcox JE. Left Ventricular Assist Device Management in the Emergency Department. *West J Emerg Med*. 2018;19(5):834-841.](http://paperpile.com/b/1qDVSL/inSa)


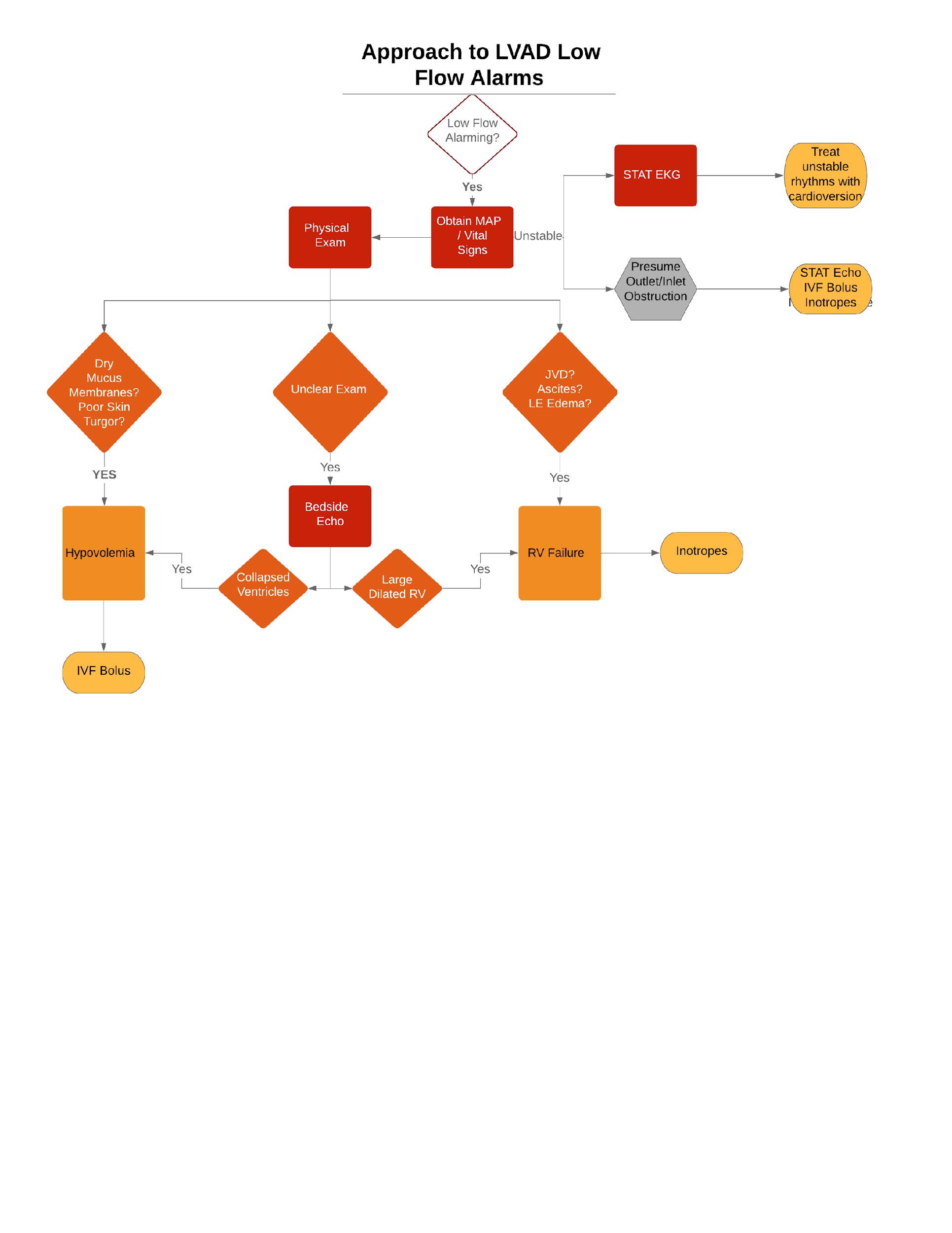


Note: *EKG*, electrocardiography; *MAP*, mean arterial pressure; *IVF*, intravenous fluids; *JVD*, jugular vein distention; *LE*, lower extremity; *RV*, right ventricle.

Source: Author owned, adapted and modified from [Trinquero P, Pirotte A, Gallagher LP, Iwaki KM, Beach C, Wilcox JE. Left Ventricular Assist Device Management in the Emergency Department. *West J Emerg Med*. 2018;19(5):834-841.](http://paperpile.com/b/1qDVSL/inSa)

Quick Summary Table

| LVAD Conditions and Complications | | | |
| --- | --- | --- | --- |
| Physiological Problem | Consideration and Differential | Evaluation | Management |
| Decreased Preload | - Hypovolemia (bleeding, dehydration, septic shock)  - RV Failure  - LVAD Failure (inflow cannula or mechanical obstruction)  - Suction Event | Cardiac POCUS  - Small RV suggests low preload  - Small LV suggests suction event  - Large RV and small LV suggests RV failure  - Large RV and LV suggests pump thrombosis/obstruction | - Hypovolemia: Provide IVFs or blood  - RV Failure: Provide vasopressor/inotropes, pulmonary vasodilators  - Inflow/mechanical obstruction due to thrombosis: anticoagulate |
| Increased Afterload | Inability to empty the LVAD chamber: hypertensive event, outflow cannula obstruction (thrombosis/mechanical kink) | Cardiac POCUS | Reduce blood pressure with vasodilators to MAP < 80 mmHg |
|  | | | |
| LVAD Complications | Alarm/Notification | Considerations | Management |
| Pump Failure | “Low Flow – Call Hospital Contact” (Red Visual Signal) | Evaluate Connections to battery and the battery | Emergent LVAD consultation. Reconnect any disconnections and ensure battery is inserted |
| Power Disruption | “Connect Power Immediately” (Yellow/Red Battery Icon) | Evaluate all connections (including controller-driveline, controller-power supply) | Emergent LVAD consultation. If connections are intact with charged device, exchange of the device may be required. |
| Driveline Damage/Electrical Fault | “Connect Driveline” (Red Visual Signal) | Driveline contains 6 separate wires with redundancy, evaluate all lines for damage | Emergent LVAD consultation. Patients with cardiogenic shock require resuscitation with vasopressor and/or inotropes. |
| High Flow | High Flow Alarm | High flow and normal watts suggest infection/sepsis due to peripheral vasodilation. | Closely evaluate for sepsis, treat with antibiotics/vasopressors. May require CTS consultation. |
| Low Flow | Low Flow Alarm – Assess clinical stability and perfusion status.  Evaluate pump function and components, examine for hypovolemia, thrombosis, dysrhythmia (VT/VF). | Evaluate pump and connections  Obtain ECG and POCUS  - collapsed IVC suggests decreased preload, high RV:LV ratio suggests RV dysfunction  Laboratory assessment with CBC, haptoglobin, LDH, urinalysis  Obtain IV access bilaterally | Anticoagulate for thrombosis  Vasopressors/inotropes may be needed  IVF resuscitation  Pulmonary vasodilators  Cardioversion |

Source: Author owned, but modified and adopted from [Long B, Robertson J, Koyfman A, Brady W. Left ventricular assist devices and their complications: A review for emergency clinicians. *Am J Emerg Med*. 2019;37(8):1562-1570.](http://paperpile.com/b/1qDVSL/oZqj)

References:

1. Rudolph JW, Simon R, Raemer DB, Eppich WJ. Debriefing as formative assessment: closing performance gaps in medical education. *Acad Emerg Med.* 2008;15(11):1010-1016.
2. Zinoviev R, Lippincott CK, Keller SC, Gilotra NA. In Full Flow: Left Ventricular Assist Device Infections in the Modern Era. *Open Forum Infect Dis.* 2020;7(5):ofaa124.
