# Supplementary material for: Left Ventricular Assist Device Multialarm Emergency: A High-Fidelity Simulation Case for Emergency Medicine Residents
Source: MedEdPORTAL. 2021 May 5;17:11156. doi: 10.15766/mep_2374-8265.11156 (PMC8096883; doi:10.15766/mep_2374-8265.11156)
Supplement: Supplementary file 1 — Institutional LVAD Coordinator Educational Presentation.pptxHeartMate 3 Task Trainer Setup.docxSimulation Case.docxSimulation Images.docxCritical Actions.docxDebriefing Materials.docxSurvey.docx [file mep_2374-8265.11156-s001.zip › G. Survey.docx]

Appendix G: Survey

**LVAD Catastrophe Simulation Evaluation Form**

|  | Strongly  Disagree | Disagree | Neutral | Agree | Strongly  Agree |
| --- | --- | --- | --- | --- | --- |
| 1. This case presented during the simulation is relevant to my work. | 1 | 2 | 3 | 4 | **5** |
| 1. The simulation case was realistic. | 1 | 2 | 3 | 4 | **5** |
| 1. This simulation case was effective in teaching basic resuscitation skills. | 1 | 2 | 3 | 4 | **5** |
| 1. The debrief promoted reflection and team discussion. | 1 | 2 | 3 | 4 | **5** |
| 1. The group discussion helped me develop and prioritize evaluation and management options for a unstable LVAD patient. | 1 | 2 | 3 | 4 | **5** |
| 1. The facilitators created a safe environment for discussion and exploration. | 1 | 2 | 3 | 4 | **5** |

After participating in this session, how confident are you in your ability to:

|  | Very Unconfident | Unconfident | Neutral | Confident | Very Confident |
| --- | --- | --- | --- | --- | --- |
| Demonstrate ability to assess an LVAD patient. | 1 | 2 | 3 | 4 | **5** |
| Obtain a blood pressure on an LVAD patient. | 1 | 2 | 3 | 4 | **5** |
| Troubleshoot an LVAD and its various alarms. | 1 | 2 | 3 | **4** | 5 |
| Run ACLS, operate a defibrillator, and deliver the appropriate therapy in an LVAD patient in cardiac arrest. | 1 | 2 | 3 | 4 | **5** |
| Formulate a list of possible diagnoses and prioritize elements of evaluation. | 1 | 2 | 3 | 4 | **5** |
| Manage septic shock in an acutely ill patient. | 1 | 2 | 3 | 4 | **5** |
| Inform appropriate consultants and construct a disposition plan. | 1 | 2 | 3 | 4 | **5** |

Can you list/describe one or more ways this session will change how you do your job?

How could we improve this simulation and workshop?

Additional Comments:
