# Supplementary material for: Left Ventricular Assist Device Multialarm Emergency: A High-Fidelity Simulation Case for Emergency Medicine Residents
Source: MedEdPORTAL. 2021 May 5;17:11156. doi: 10.15766/mep_2374-8265.11156 (PMC8096883; doi:10.15766/mep_2374-8265.11156)
Supplement: Supplementary file 1 — Institutional LVAD Coordinator Educational Presentation.pptxHeartMate 3 Task Trainer Setup.docxSimulation Case.docxSimulation Images.docxCritical Actions.docxDebriefing Materials.docxSurvey.docx [file mep_2374-8265.11156-s001.zip › B. HeartMate 3 Task Trainer Setup.docx]

Appendix B – Integration of HeartMate III task trainer

Figure 1: LVAD HeartMate 3 task trainer components (battery packs, controller, driveline, and pump with closed-loop)


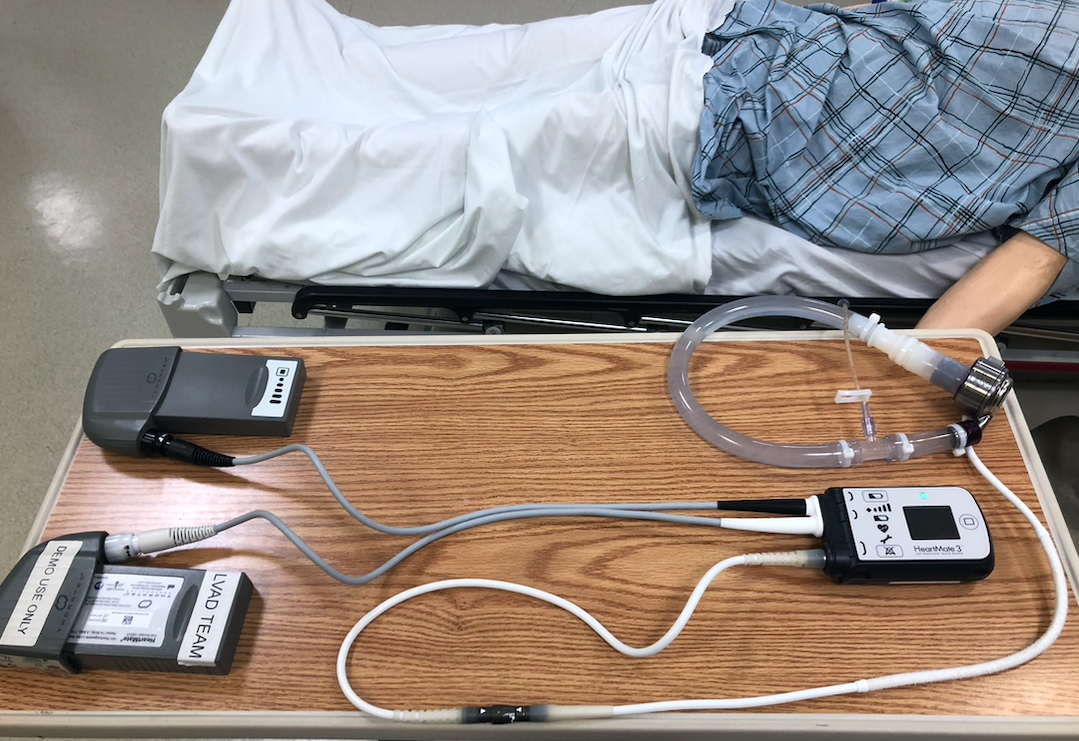


Source: Author-owned

Figure 2: LVAD HeartMate 3 Controller and Battery Pack with Driveline inserted through the abdomen


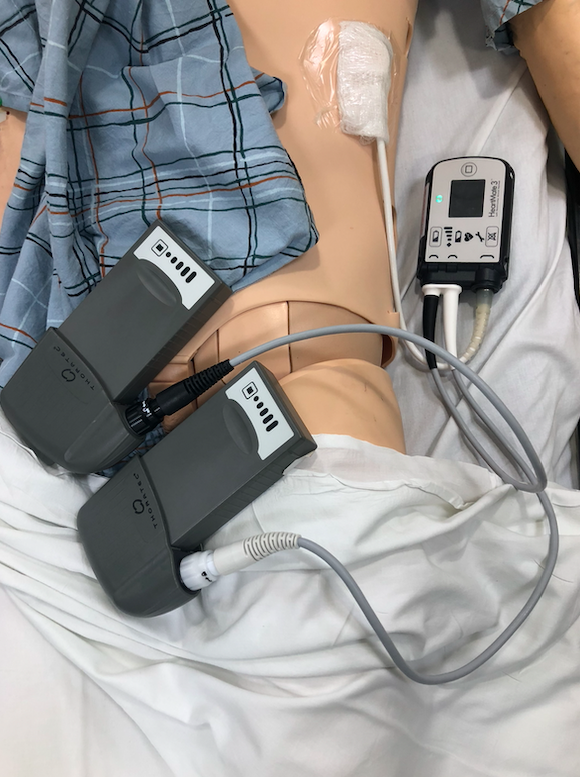


Source: Author-owned

Figure 3: HeartMate 3 task trainer pump placed under chest plate of SimMan 3G.


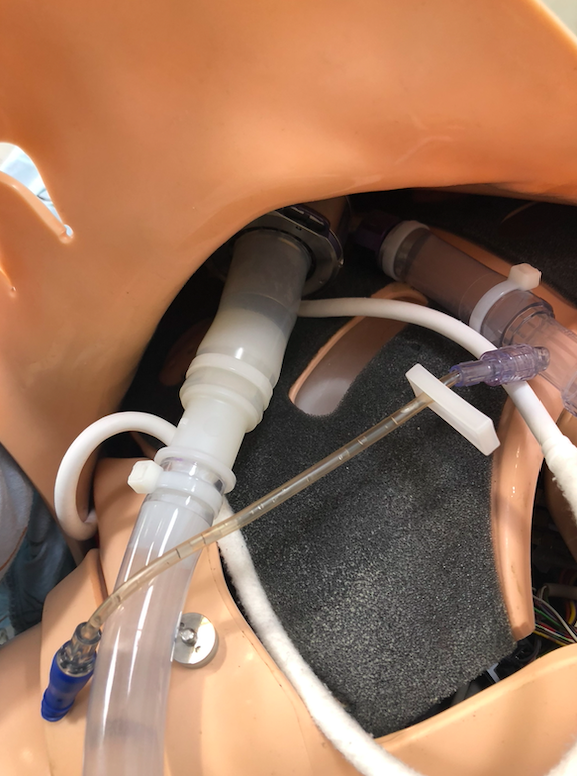


Source: Author-owned
