# Supplementary material for: Left Ventricular Assist Device Multialarm Emergency: A High-Fidelity Simulation Case for Emergency Medicine Residents
Source: MedEdPORTAL. 2021 May 5;17:11156. doi: 10.15766/mep_2374-8265.11156 (PMC8096883; doi:10.15766/mep_2374-8265.11156)
Supplement: Supplementary file 1 — Institutional LVAD Coordinator Educational Presentation.pptxHeartMate 3 Task Trainer Setup.docxSimulation Case.docxSimulation Images.docxCritical Actions.docxDebriefing Materials.docxSurvey.docx [file mep_2374-8265.11156-s001.zip › C. Simulation Case.docx]

**Appendix C**

| **MedEdPORTAL Simulation Case Template**  **SIMULATION CASE TITLE:** Left Ventricular Assist Device Multi-Alarm Emergency  **AUTHORS:** Ryan Barnicle MD, M.Ed, Sean Boaglio DO, Scott Johnson MD  **LEARNER AUDIENCE:** EM Residents (PGY1, PGY2, PGY3) | |
| --- | --- |
| **PATIENT NAME:** Arthur Morgan  **PATIENT AGE:** 62  **CHIEF COMPLAINT:** malaise, vomiting, diarrhea  **PHYSICAL SETTING:** community ED resuscitation room, > 60 minutes from LVAD tertiary care center | |
|  | |
| **Brief narrative description of case** | A 65-year-old male presents to a community emergency department with worsening lethargy and ill-appearance. EMS was unable to obtain a blood pressure in the field. The spouse states that he has also had diarrhea and vomiting for the past 24 hours. She notes he has had an LVAD for about six months and follows with an affiliated tertiary care center several hours away. On arrival, the LVAD alarm will start going off.  The physician team will need to immediately evaluate LVAD function and assess vital signs appropriately and will discover the patient has a low MAP and a low-grade fever. The alarming controller will also need to  be evaluated. All alarms (battery and low flow) will need to be addressed with acute interventions. The LVAD Coordinator Team will need to be contacted for management recommendations and disposition.  Despite appropriate interventions, the patient will progress to a PEA arrest requiring resuscitation by ACLS protocols. Once underlying sepsis and hypovolemia is treated, the patient will obtain ROSC. Intubation and transfer will be required. |
| **Goal and Learning Objectives** | Goal:  To provide emergency medicine resident physicians with an authentic, high-fidelity simulation in managing the critically ill patient with an alarming left ventricular assist device (LVAD)  Objectives:  -By the end of the simulation exercise, all learners should be capable of completing the following objectives   1. - Demonstrate a systematic approach to evaluating a patient with an LVAD 2. - Attain an accurate blood pressure in a patient with an LVAD 3. - Differentiate and rectify various LVAD alarms, including impending power failure 4. - Formulate a differential diagnosis for LVAD “Low Flow” alarms 5. - Diagnose and manage septic shock in a patient with an LVAD 6. - Summarize a concise and relevant report to the LVAD consultant team 7. - Appropriately employ ACLS for a patient with an LVAD in cardiac arrest |
| **Critical Actions** | - Confirm LVAD is functioning by assessing for “thrum” under chest wall  - Obtain a reliable blood pressure with either doppler or arterial line  - Identify a fever via core temperature  - LVAD coordinator contact was attempted and reattempted until completed  - Ensure a reliable source of power when the battery alarm starts going off  - Treat septic shock and hypovolemia empirically once identified as the likely source of the low flow alarm with antibiotics and fluid resuscitation and vasopressors  - Inspect driveline insertion site in sterile fashion  - Give appropriate chest compressions during cardiac arrest  - Perform intubation in a hemodynamically neutral fashion |
| **Learner Preparation or Prework** | Our learners underwent an overview (Appendix A) from one of our institution’s LVAD coordinators at the weekly conference prior to the simulation. This included an explanation of heart failure patients who qualify for LVAD placement, preferred bloodwork to be ordered in the ED (including and emphasis on LDH to assess for hemolysis), how to assess blood pressure, speed / power / flow displays, hazard and advisory alarm management, driveline infection assessment, LVAD anticoagulation guidelines, common complications, and cardiac arrest management.  At the time of the simulation, learners should be informed that this is a safe environment intended to improve learner confidence and patient care. The simulation experience is intended to be formative and is solely for the purpose of practice and learning. |

| **Initial Presentation** | | | |
| --- | --- | --- | --- |
| **Initial vital signs** | HR 80 bpm, BP --/-- mmHg, RR 20 bpm, SpO2 96% RA, T: 37.5º C orally, Fingerstick: 157, GCS:  13  (E3  V4  M6) | | |
| **Overall Setting and Appearance** | Learners see an ill-appearing, lethargic middle-aged male responsive to verbal stimuli. He is connected to an LVAD HeartMate III that is at his side. | | |
| **Confederates (e.g., standardized participants) and their roles in the room at case start** | - The Chief Resident or extra Attending will perform the role of Paramedic / Nurse / Wife in order to provide background information (HPI and ROS) and necessary stimulus. He/She may be needed to subtly stimulate LVAD alarms. “EMS” will explain they were unable to get blood pressure and thus came immediately to the nearest hospital. They provide LVAD card and spare power packs. - The Team Leader should be a Senior Resident who already has comfort with basic resuscitation of the undifferentiated patient in shock. - Other residents will be directed to handle the tasks of obtaining intravenous access, administering medication, and managing the airway. - The role of the LVAD Coordinator can be voiced by the Attending running the simulation session via the phone or intercom. | | |
| **HPI / ROS** | The patient’s wife called 911 prior to arrival because the patient appeared very tired and unwell, had decreased responsiveness, and felt “hot.” He has been having vomiting and diarrhea for about one day with nearly no eating or drinking. He was treated with Augmentin about one week ago for sinusitis and felt better after.  General: +fatigue, +weakness, +weight loss, +chills  Neurologic: no focal weakness, no slurred speech, no headaches  Cardiovascular: no chest pain, +lightheadedness  Respiratory: no dyspnea, no cough  Gastrointestinal: +abdominal pain, +vomiting, +diarrhea, +poor oral intake  Genitourinary: no dysuria, +decreased urine  Integumentary: no rash, no jaundice | | |
| **Past Medical/Surgical History** | **Medications** | **Allergies** | **Family History / Social History** |
| Heart failure s/p LVAD, Hypertension, Hyperlipidemia, Diabetes Mellitus II, Chronic Kidney Disease | warfarin, metformin, lisinopril, aspirin, metoprolol, atorvastatin | sulfa drugs | Father: Died from MI, Mother: Lung cancer  Lives with wife, retired police officer, former smoker, no current alcohol/drugs |
| **Physical Examination** | | | |
| **General** | toxic appearing | | |
| **HEENT** | dry mucous membranes | | |
| **Neck** | No JVD | | |
| **Lungs** | normal chest excursion, lungs clear to auscultation bilaterally | | |
| **Cardiovascular / Chest Wall** | continuous LVAD thrum palpated and auscultated, no pulses palpable | | |
| **Abdomen** | soft, mildly distended, mildly tender in all quadrants, sterile driveline dressing in upper left quadrant clean/dry/intact *(possible cellulitis and purulence underneath if desired)* | | |
| **Neurological** | easily arousable to voice but sleepy, speaks clearly, confused to some questions, no focal deficits | | |
| **Skin** | pale, diaphoretic | | |
| **GU** | Unremarkable | | |

| **Cast and Realism** | | |
| --- | --- | --- |
| Patient: Computerized Mannequin - SimMan 3G | | |
| Device: HeartMate 3 “loop” trainer with HeartMate 3 controller | | |
| **Required Monitors** | | |
| EKG Leads/Wires | Temperature Probe | Capnography |
| NIBP Cuff | Doppler | Defibrillator Pads |
| Pulse Oximeter | Arterial Line | Other: |
| **Required Equipment** | | |
| Gloves, Mask, Gowns | Stethoscope | Airway Box |
| Nasal Cannula | Non-Rebreather Mask | Bag Valve Mask |
| Defibrillator | Arterial Line Kit | Sterile Dressing Kit |
| IV Bags/Lines | IV Medications / Code Cart | Phone |
| **Moulage** | | |
| Driveline should exit abdomen, be covered with dressing *(optional cellulitis moulage with purulent material underneath dressing if desired).* See Figure 2. | | |

| **Instructor Notes - Changes and CASE Branch Points** | | |
| --- | --- | --- |
| **Intervention / Time point** | **Change in Case** | **Additional Information** |
| *Team enters room and begins assessment by connecting patient to monitors and interviewing patient/EMS/wife* | *Patient is initially able to answer some questions but appears groggy, unreliable. Vomiting sounds should occur.* | *Team should note that the patient is not connected to the monitor and has an LVAD controller at his side.* |
| *LVAD thrum assessed by one of the team members* | *If the chest is not palpated for functioning LVAD, patient should have PEA arrest.* | *Paramedic can say, “I don’t know much about these LVADs but I felt it working through his chest wall in the ambulance.” The medic can provide LVAD coordinator card to team.* |
| *1 minute into the case: LVAD begins to display/sound alarm “Connect Power”* | *This will be very distracting and the case cannot proceed without intervention.*  *Once new batteries are connected, the alarm will cease.* | *Paramedic indicates the wife sent spare batteries with ambulance, which must be connected by residents to stop the alarm. When “Connect Power” alarm sounds, new batteries should be connected. If not, PEA arrest happens in 3 minutes. LVAD thrum will cease.* |
| *3 minutes into the case* | *BP is unavailable by non-invasive measurement and will remain so without doppler or arterial line, the patient will have PEA cardiac arrest if not obtained.* | *RN alerts the provider: “Doctors, shouldn’t we use doppler to get a MAP?” MAP will be 55 or BP will be 82/40, which is too low for LVAD normal function.* |
| *5 minutes into case:* | *LVAD coordinators must be contacted. First attempt will fail.*  *Rectal temperature should be obtained to find fever. Additional tests can be requested as below.*  *[ ] Labs: CBC, BMP, PT/INR, LDH, Troponin, BNP, Lactate*  *[ ] Rectal temperature*  *[ ] ECG*  *[ ] CXR*  *[ ] POCUS echo* | *Team will need to continue empiric resuscitation with IVFs, antibiotics.*  *[ ] IVF bolus*  *[ ] norepinephrine gtt*  *[ ] empiric antibiotics* |
| *10 minutes into case: Despite appropriate actions, the LVAD controller will begin to show LOW FLOW alarm due to persistent hypotension.* | *The differential for flow alarms is extensive but the team should conclude that a combination of septic shock and volume loss are contributing to hypotension.* | *When “Low Flow” alarm sounds, differential will include sepsis and volume depletion given story of gastroenteritis. VAD thrombus or suction event can be considered as well. Team can silence the alarm temporarily.* |
| *12 minutes into case: Inevitable PEA arrest. Patient will become unresponsive. Arterial BP will be lost.* | *[ ] re-auscultate chest to confirm LVAD is functioning*  *[ ] start chest compressions*  *[ ] administer 1 mg epinephrine every 5 minutes*  *[ ] give additional bolus of crystalloid IVF* | *Blood work will result:*  *CBC - WBC 16.2, HGB 10.5, HCT 38%, PLT 345*  *BMP - Na 134, Cl 108, K 5.6, HCO3 10, Cr 3.02, BUN 40, Glu 165, Ca 8.2*  *Troponin - 0.17*  *BNP – 4020*  *Lactate - 5.2*  *LDH – 220*  *INR – 2.6*  *IVFs / epinephrine given →  ROSC* |
| *20 minutes into case*  *Post-ROSC*  *Rhythm: NSR*  *HR: 90*  *BP:  105/75 (arterial)*  *MAP: 66 (doppler)*  *RR:  16*  *O_2_SAT: 95% on 40% FiO2*  *T:  38.5^o^C (rectal)* | *Patient remains minimally responsive but is moving all extremities. LVAD appears to now be functioning.*  *[ ] intubate for airway protection*  *[ ] vasopressors continued*  *[ ] call VAD coordinator again* | *Scenario ends when transfer is arranged with VAD coordinator at tertiary care center* |

**Ideal Scenario Flow**

*The learners enter the room to find an ill-appearing male with a recent LVAD implantation. The patient can answer some questions but collateral information is filled in by the paramedic, who is unfamiliar with LVADs and expresses concern over unobtainable blood pressure. While the HPI is being taken, the battery connect alarm begins to sound as the batteries connected are dead. The team must replace these with the patient’s spare batteries immediately. The patient is then found to be hypotensive by either doppler or arterial line. The team discovers an occult fever via rectal temperature assessment. Given likelihood of driveline infection, this is inspected in sterile fashion and appears to be clean. Given the constellation of fever, hypotension, and report of volume loss the team begins to treat for septic/hypovolemic shock empirically but the patient continues to deteriorate with further depressed mental status. Labs are consistent with shock state, including elevated lactate and signs of renal failure. Given normal CBC, GI bleed is not suspected. Given adequate INR, LVAD pump thrombosis is not treated with anticoagulation. The LVAD coordinator is unavailable initially. Despite fluids and antibiotics, the patient progresses to PEA arrest. Chest compressions should NOT be held just because he has an LVAD. Standard ACLS occurs, including the administration of epinephrine. ROSC is obtained after one round of ACLS and the patient is started on norepinephrine infusion. Intubation proceeds because the patient has a depressed mental status but appears otherwise neurologically intact after ROSC. The LVAD coordinator calls back and agrees to transfer to the LVAD center now that the patient seems stable on vasopressor support.*

**Anticipated Management Mistakes**

1. *Team does not use doppler or arterial line to obtain reliable blood pressure: While our residents are familiar with arterial line placement, using doppler to assess for MAP is not common for non-LVAD patients. We found that leaving the doppler equipment at the bedside prompted its use.*
2. *Failure to consult LVAD coordinator: Regardless of stability, LVAD coordinators must always be consulted for appropriate treatment and disposition.*
3. *Withholding CPR once PEA arrest occurs: While manufacturer guidelines may warn against chest compressions, current guidelines still advocate that ACLS follows standard algorithms while underlying cause of arrest is treated. In this case, the shock state must be addressed.*
